# Supplementary material for: MetaRibo-Seq measures translation in microbiomes
Source: Nat Commun. 2020 Jun 29;11:3268. doi: 10.1038/s41467-020-17081-z (PMC7324362; doi:10.1038/s41467-020-17081-z)
Supplement: Supplementary file 10 — Supplementary Data 7 [file 41467_2020_17081_MOESM10_ESM.zip › File2/Confidence_VeryHigh_Taxonomy/64214_out.krona.html]

Javascript must be enabled to view this page.

members
magnitude
magnitudeUnassigned
count
unassigned
taxon
rank

64214\_out

4

1

SRS078242\_contig\_number\_contig-100\_30484.68105

3
superkingdom
2

phylum
3
1239

class
3
186801

3
order
186802

family
3
541000

216851
3
genus

1946510

SRS018541\_contig\_number\_11239SRS147557\_contig\_number\_24663SRS148196\_contig\_number\_16556
species
3
